# *catena*-Poly[ammonium (cadmium-tri-μ-thio­cyanato-κ^4^ *S*:*N*;κ^2^ *N*:*S*)–1,4,10,13,16-hexa­oxa­cyclo­octa­decane (1/1)]

**DOI:** 10.1107/S1600536812004898

**Published:** 2012-02-24

**Authors:** V. Ramesh, K. Rajarajan, K. Sendil Kumar, A. Subashini, M. NizamMohideen

**Affiliations:** a Research and Development Centre, Bharathiyar University, Coimbatore 641 046, India; b Department of Physics, Rajeswari Vedachalam Government Arts College, Chengalpet 603 001, India; c Department of Physics, The New College (Autonomous), Chennai 600 014, India

## Abstract

In the title compound, {(NH_4_)[Cd(NCS)_3_]·C_12_H_24_O_6_}_*n*_, the Cd^2+^ ion, the ammonium cation, one of the SCN^−^ ligands and the macrocycle are located on mirror planes. The thiocyanate anions act as bridging ligands between the Cd^II^ ions, leading to a polymeric chain arrangement extending along [001] around a twofold screw axis. The ammonium ions are contained within the bowl of the macrocycle *via* extensive N—H⋯O hydrogen bonding.

## Related literature

For a singly bridged cadmium thio­cyanate complex, see: Bose *et al.* (2004 ▶). For a triply bridged cadmium thio­cyanate complex, see: Chen *et al.* (2002 ▶). For an S-bound terminal thio­cyanate cadmium complex, see: Nfor *et al.* (2006 ▶). For polymeric structures of complexes, see: Lobana *et al.* (2008 ▶). For the structures and properties of cadmium compounds, see: Gu *et al.* (2011 ▶); Zheng *et al.* (2004 ▶); Rajesh *et al.* (2004 ▶). For bond lengths and angles of related compounds, see: Nawaz *et al.* (2010 ▶).
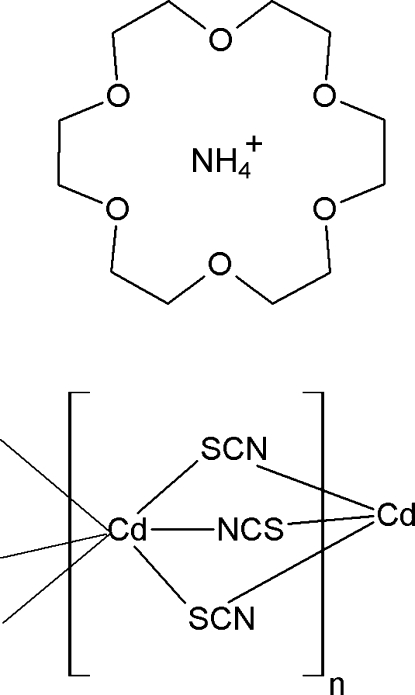


## Experimental

### 

#### Crystal data

(NH_4_)[Cd(NCS)_3_]·C_12_H_24_O_6_
*M* *_r_* = 568.99
Orthorhombic, 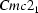

*a* = 14.7568 (6) Å
*b* = 15.4378 (6) Å
*c* = 10.6383 (5) Å
*V* = 2423.54 (18) Å^3^
*Z* = 4
Mo *K*α radiation
μ = 1.20 mm^−1^
*T* = 293 K
0.30 × 0.25 × 0.20 mm

#### Data collection

Bruker Kappa APEXII CCD diffractometer
Absorption correction: multi-scan (*SADABS*; Sheldrick, 2004 ▶) *T*_min_ = 0.716, *T*_max_ = 0.796
11323 measured reflections
2483 independent reflections
2445 reflections with *I* > 2σ(*I*)
*R*_int_ = 0.019

#### Refinement

*R*[*F* ^2^ > 2σ(*F* ^2^)] = 0.013
*wR*(*F* ^2^) = 0.034
*S* = 1.09
2483 reflections
154 parameters
5 restraints
H atoms treated by a mixture of independent and constrained refinement
Δρ_max_ = 0.20 e Å^−3^
Δρ_min_ = −0.36 e Å^−3^
Absolute structure: Flack (1983 ▶), 7607 Friedel pairs
Flack parameter: 0.005 (15)

### 

Data collection: *APEX2* (Bruker, 2004 ▶); cell refinement: *APEX2* and *SAINT* (Bruker, 2004 ▶); data reduction: *SAINT* and *XPREP* (Bruker, 2004 ▶); program(s) used to solve structure: *SHELXS97* (Sheldrick, 2008 ▶); program(s) used to refine structure: *SHELXL97* (Sheldrick, 2008 ▶); molecular graphics: *ORTEP-3* (Farrugia, 1997 ▶) and *Mercury* (Macrae *et al.*, 2006 ▶); software used to prepare material for publication: *WinGX* (Farrugia, 1999 ▶), *PLATON* (Spek, 2009 ▶) and *publCIF* (Westrip, 2010 ▶).

## Supplementary Material

Crystal structure: contains datablock(s) global, I. DOI: 10.1107/S1600536812004898/zb2021sup1.cif

Structure factors: contains datablock(s) I. DOI: 10.1107/S1600536812004898/zb2021Isup2.hkl

Additional supplementary materials:  crystallographic information; 3D view; checkCIF report

## Figures and Tables

**Table 1:** Hydrogen-bond geometry (Å, °)

*D*—H⋯*A*	*D*—H	H⋯*A*	*D*⋯*A*	*D*—H⋯*A*
N3—H3*E*⋯O2	0.89 (1)	2.03 (1)	2.9130 (19)	174 (3)
N3—H3*D*⋯O4	0.90 (1)	2.05 (3)	2.892 (3)	155 (5)

**Fig. 1.**
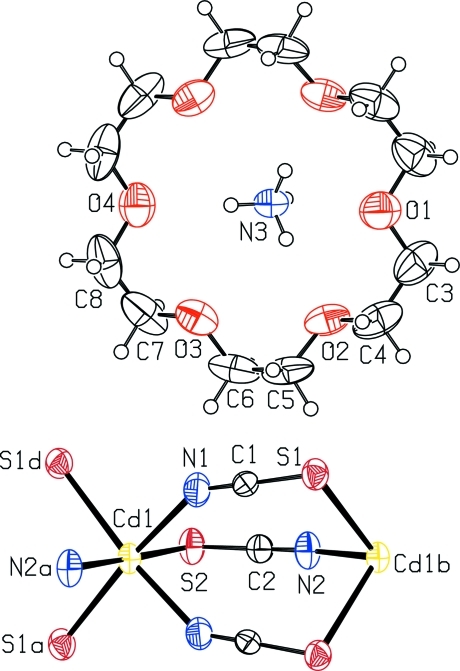
The molecular structure of the title compound with the atom numbering scheme and 50% probability displacement ellipsoids. H atoms are presented as a small spheres of arbitrary radius.

**Fig. 2.**
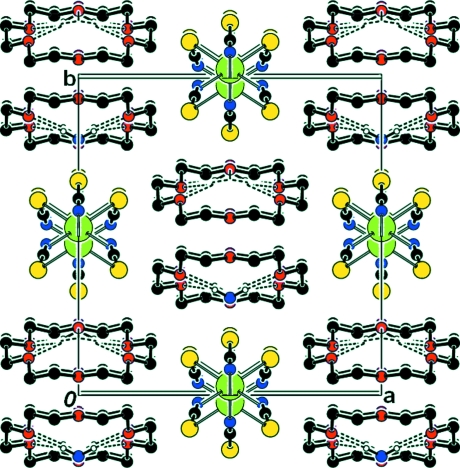
Molecular packing, viewed down c axis.

## supplementary crystallographic information

### Comment

Thiocyanate anion is known to bind the cadmium ion in different modes: terminal N-bound, terminal S-bound (Nfor *et al.* 2006) or N:*S*-bridging ligand. As a bridging ligand, it may give rise to a singly bridged (Bose *et al.* 2004), doubly bridged or triply bridged (Chen *et al.* 2002) cadmium complex. Cadmium(II) complexes with thiones possess a variety of structures ranging from four- to six-coordinate species with tetrahedral and octahedral environments for the CdII atom, respectively. In some cases, these units further aggregate to form polymeric structures (Lobana *et al.*, 2008). The interest in cadmium compounds was provoked by their luminescent properties (Zheng *et al.*, 2004), magnetic and catalytic properties (Gu *et al.*, 2011) and non-linear optical properties (Rajesh *et al.*, 2004). Herein, we report the synthesis and crystal structure of cadmium complex, the title compound, (I), coordinated by nitrogen and sulfur.

A perspective view of compound (I) with the atom-numbering scheme is shown in Fig. 1. The Cd^II^ ions are bridged by a pair of thiocyanate N:*S*-bridging ligands around a twofold screw axis. Two *trans*-N:*S*-bridging thiocyanates complete the N3S3 donor set around the Cd atom. The thiocyanate anions function as bridging ligands between the Cd^II^ centres, leading to a chain-like arrangement expanding along [001]. The thiocyanate ligands are almost linear.

The Cd—S bond lengths are 2.747 (4) and 2.728 (4) Å. These are in agreement with those reported for related compounds (Nawaz *et al.*, 2010). The bond distances of N-bonded NCS groups [Cd—N(NCS) 2.347 (4) and 2.375 (4) Å]. These values agree well with those observed in [Cd(NCS)_2_(1-vinylimidazole)_4_] (Gu *et al.*, 2011). The values of the bond angles around cadmium are close to those expected for a regular octahedral geometry, the largest angular deviation being observed for the N2 –Cd1- N1 angle [93.34 (5)°].

The parameters of hydrogen bonds are given in the Table 1. The thiocyanate anions function as bridging ligands between the CdII centres, leading to a chain-like arrangement are parallel to one another and expanding along [001]. The ammonium molecules also participate in extensive N—H···O hydrogen bonding, as shown in Fig. 2.

### Experimental

The mixture of 18-crown-6 (C_12_H_24_O_6_), CdCl_2_ and NH_4_SCN (molar ratio 1:1:3) were thoroughly dissolved in double distilled water and stirred for 5 h to obtain a homogeneous mixture. The colorless single crystals were obtained after the filtrate had been allowed to stand at room temperature for three weeks.

### Refinement

Carbon H atoms were placed geometrically (C—H = 0.97 Å) and treated as riding with *U*_iso_(H) = 1.2*U*_eq_(C). Water H atoms were located in calculated positions and treated in the subsequent refinement as riding atoms, with N—H = 0.89 Å and *U*_iso_(H) = 1.5*U*_eq_(O).

### Crystal data

**Table:**

(NH_4_)[Cd(NCS)_3_]·C_12_H_24_O_6_	*F*(000) = 1160
*M**_r_* = 568.99	*D*_x_ = 1.559 Mg m^−^^3^
Orthorhombic, *C**m**c*2_1_	Mo *K*α radiation, λ = 0.71073 Å
Hall symbol: C 2c -2	Cell parameters from 5280 reflections
*a* = 14.7568 (6) Å	θ = 2.6–26.7°
*b* = 15.4378 (6) Å	µ = 1.20 mm^−^^1^
*c* = 10.6383 (5) Å	*T* = 293 K
*V* = 2423.54 (18) Å^3^	Block, colourless
*Z* = 4	0.30 × 0.25 × 0.20 mm

### Data collection

**Table:**

Bruker Kappa APEXII CCD diffractometer	2483 independent reflections
Radiation source: fine-focus sealed tube	2445 reflections with *I* > 2σ(*I*)
Graphite monochromator	*R*_int_ = 0.019
ω and φ scan	θ_max_ = 26.0°, θ_min_ = 2.6°
Absorption correction: multi-scan (*SADABS*; Sheldrick, 2004)	*h* = −18→18
*T*_min_ = 0.716, *T*_max_ = 0.796	*k* = −19→19
11323 measured reflections	*l* = −13→13

### Refinement

**Table:**

Refinement on *F*^2^	Hydrogen site location: inferred from neighbouring sites
Least-squares matrix: full	H atoms treated by a mixture of independent and constrained refinement
*R*[*F*^2^ > 2σ(*F*^2^)] = 0.013	*w* = 1/[σ^2^(*F*_o_^2^) + (0.020*P*)^2^ + 0.1189*P*] where *P* = (*F*_o_^2^ + 2*F*_c_^2^)/3
*wR*(*F*^2^) = 0.034	(Δ/σ)_max_ = 0.003
*S* = 1.09	Δρ_max_ = 0.20 e Å^−^^3^
2483 reflections	Δρ_min_ = −0.36 e Å^−^^3^
154 parameters	Extinction correction: *SHELXL97* (Sheldrick, 2008), Fc^*^=kFc[1+0.001xFc^2^λ^3^/sin(2θ)]^-1/4^
5 restraints	Extinction coefficient: 0.0058 (2)
Primary atom site location: structure-invariant direct methods	Absolute structure: Flack (1983), 7607 Friedel pairs
Secondary atom site location: difference Fourier map	Flack parameter: 0.005 (15)

### Special details

**Table:**

Geometry. All esds (except the esd in the dihedral angle between two l.s. planes) are estimated using the full covariance matrix. The cell esds are taken into account individually in the estimation of esds in distances, angles and torsion angles; correlations between esds in cell parameters are only used when they are defined by crystal symmetry. An approximate (isotropic) treatment of cell esds is used for estimating esds involving l.s. planes.
Refinement. Refinement of F^2^ against ALL reflections. The weighted R-factor wR and goodness of fit S are based on F^2^, conventional R-factors R are based on F, with F set to zero for negative F^2^. The threshold expression of F^2^ > 2sigma(F^2^) is used only for calculating R-factors(gt) etc. and is not relevant to the choice of reflections for refinement. R-factors based on F^2^ are statistically about twice as large as those based on F, and R- factors based on ALL data will be even larger.

### Fractional atomic coordinates and isotropic or equivalent isotropic displacement parameters (Å^2^)

**Table:**

	*x*	*y*	*z*	*U*_iso_*/*U*_eq_	
C1	0.61632 (10)	1.06679 (10)	0.72484 (14)	0.0337 (3)	
C2	0.5000	0.87710 (13)	0.7689 (2)	0.0339 (4)	
C3	0.9203 (2)	0.92459 (19)	1.0905 (3)	0.0905 (9)	
H3A	0.9205	0.9648	1.1606	0.109*	
H3B	0.9177	0.8662	1.1240	0.109*	
C4	0.83962 (17)	0.94095 (17)	1.0097 (4)	0.0885 (10)	
H4A	0.7851	0.9382	1.0604	0.106*	
H4B	0.8436	0.9985	0.9736	0.106*	
C5	0.76317 (15)	0.89705 (16)	0.8297 (3)	0.0811 (8)	
H5A	0.7709	0.9545	0.7945	0.097*	
H5B	0.7062	0.8959	0.8751	0.097*	
C6	0.76078 (16)	0.83248 (19)	0.7272 (3)	0.0845 (9)	
H6A	0.7596	0.7745	0.7621	0.101*	
H6B	0.7065	0.8405	0.6770	0.101*	
C7	0.8399 (2)	0.78484 (18)	0.5498 (3)	0.0923 (10)	
H7A	0.7847	0.7908	0.5010	0.111*	
H7B	0.8430	0.7259	0.5811	0.111*	
C8	0.9199 (2)	0.80317 (17)	0.4690 (2)	0.0921 (10)	
H8A	0.9185	0.7664	0.3952	0.110*	
H8B	0.9186	0.8631	0.4415	0.110*	
N1	0.60575 (11)	1.03455 (9)	0.62874 (16)	0.0496 (4)	
N2	0.5000	0.91401 (12)	0.86273 (19)	0.0454 (5)	
O1	1.0000	0.93509 (16)	1.0190 (3)	0.0776 (8)	
O2	0.83461 (10)	0.87912 (10)	0.91271 (18)	0.0692 (4)	
O3	0.83851 (11)	0.84297 (10)	0.65100 (18)	0.0703 (4)	
O4	1.0000	0.78737 (15)	0.5382 (2)	0.0742 (7)	
N3	1.0000	0.80499 (15)	0.8089 (2)	0.0476 (5)	
Cd1	0.5000	0.971763 (8)	0.494929 (17)	0.03587 (6)	
S1	0.63319 (3)	1.11267 (3)	0.86256 (4)	0.04216 (10)	
S2	0.5000	0.82400 (4)	0.63561 (5)	0.04418 (14)	
H3E	0.9497 (12)	0.8303 (18)	0.836 (3)	0.096 (10)*	
H3C	1.0000	0.7522 (12)	0.844 (3)	0.076 (11)*	
H3D	1.0000	0.817 (4)	0.7264 (16)	0.13 (2)*	

### Atomic displacement parameters (Å^2^)

**Table:**

	*U*^11^	*U*^22^	*U*^33^	*U*^12^	*U*^13^	*U*^23^
C1	0.0298 (7)	0.0389 (8)	0.0324 (7)	−0.0048 (6)	0.0026 (5)	0.0036 (6)
C2	0.0389 (11)	0.0289 (9)	0.0339 (12)	0.000	0.000	0.0067 (9)
C3	0.094 (2)	0.0801 (18)	0.097 (2)	0.0021 (14)	0.0308 (17)	−0.0239 (16)
C4	0.0702 (14)	0.0716 (13)	0.124 (3)	0.0100 (11)	0.035 (2)	−0.025 (2)
C5	0.0397 (11)	0.0731 (14)	0.130 (3)	0.0164 (10)	0.0147 (13)	0.0227 (16)
C6	0.0411 (11)	0.0820 (16)	0.130 (3)	−0.0007 (11)	−0.0205 (13)	0.0190 (17)
C7	0.097 (2)	0.0739 (16)	0.106 (2)	0.0214 (15)	−0.0549 (19)	−0.0204 (15)
C8	0.149 (3)	0.0678 (14)	0.0592 (19)	0.0343 (17)	−0.0297 (17)	−0.0138 (11)
N1	0.0539 (9)	0.0612 (9)	0.0337 (8)	−0.0148 (6)	0.0025 (7)	−0.0026 (7)
N2	0.0682 (13)	0.0370 (9)	0.0309 (9)	0.000	0.000	0.0015 (9)
O1	0.0683 (13)	0.0780 (14)	0.086 (2)	0.000	0.000	−0.0160 (14)
O2	0.0498 (8)	0.0570 (8)	0.1009 (13)	0.0127 (6)	0.0144 (8)	0.0011 (8)
O3	0.0628 (9)	0.0609 (8)	0.0872 (12)	0.0032 (7)	−0.0184 (8)	0.0017 (8)
O4	0.0963 (18)	0.0630 (13)	0.0632 (14)	0.000	0.000	−0.0012 (10)
N3	0.0385 (12)	0.0456 (12)	0.0587 (15)	0.000	0.000	0.0006 (10)
Cd1	0.04531 (9)	0.03708 (8)	0.02522 (8)	0.000	0.000	−0.00054 (7)
S1	0.0465 (2)	0.0454 (2)	0.0346 (2)	−0.00946 (16)	0.00023 (17)	−0.00481 (17)
S2	0.0649 (4)	0.0355 (3)	0.0322 (3)	0.000	0.000	−0.0024 (2)

### Geometric parameters (Å, º)

**Table:**

C1—N1	1.148 (2)	C7—C8	1.488 (4)
C1—S1	1.6463 (15)	C7—H7A	0.9700
C2—N2	1.149 (3)	C7—H7B	0.9700
C2—S2	1.638 (2)	C8—O4	1.413 (3)
C3—O1	1.410 (3)	C8—H8A	0.9700
C3—C4	1.490 (5)	C8—H8B	0.9700
C3—H3A	0.9700	N1—Cd1	2.3241 (16)
C3—H3B	0.9700	N2—Cd1^i^	2.256 (2)
C4—O2	1.408 (4)	O1—C3^ii^	1.410 (3)
C4—H4A	0.9700	O4—C8^ii^	1.413 (3)
C4—H4B	0.9700	N3—H3E	0.888 (10)
C5—O2	1.403 (3)	N3—H3C	0.897 (10)
C5—C6	1.478 (4)	N3—H3D	0.898 (10)
C5—H5A	0.9700	Cd1—N2^iii^	2.256 (2)
C5—H5B	0.9700	Cd1—N1^iv^	2.3241 (16)
C6—O3	1.414 (3)	Cd1—S2	2.7283 (6)
C6—H6A	0.9700	Cd1—S1^iii^	2.7468 (4)
C6—H6B	0.9700	Cd1—S1^v^	2.7468 (4)
C7—O3	1.402 (3)	S1—Cd1^i^	2.7468 (4)
			
N1—C1—S1	179.10 (16)	O4—C8—C7	109.28 (19)
N2—C2—S2	179.69 (19)	O4—C8—H8A	109.8
O1—C3—C4	109.6 (3)	C7—C8—H8A	109.8
O1—C3—H3A	109.7	O4—C8—H8B	109.8
C4—C3—H3A	109.7	C7—C8—H8B	109.8
O1—C3—H3B	109.7	H8A—C8—H8B	108.3
C4—C3—H3B	109.7	C1—N1—Cd1	144.86 (14)
H3A—C3—H3B	108.2	C2—N2—Cd1^i^	158.30 (17)
O2—C4—C3	110.48 (19)	C3^ii^—O1—C3	113.1 (4)
O2—C4—H4A	109.6	C5—O2—C4	111.54 (19)
C3—C4—H4A	109.6	C7—O3—C6	112.2 (2)
O2—C4—H4B	109.6	C8—O4—C8^ii^	113.4 (3)
C3—C4—H4B	109.6	H3E—N3—H3C	105 (2)
H4A—C4—H4B	108.1	H3E—N3—H3D	103 (3)
O2—C5—C6	110.46 (18)	H3C—N3—H3D	127 (5)
O2—C5—H5A	109.6	N2^iii^—Cd1—N1	93.20 (5)
C6—C5—H5A	109.6	N2^iii^—Cd1—N1^iv^	93.20 (5)
O2—C5—H5B	109.6	N1—Cd1—N1^iv^	84.36 (8)
C6—C5—H5B	109.6	N2^iii^—Cd1—S2	174.69 (5)
H5A—C5—H5B	108.1	N1—Cd1—S2	90.73 (4)
O3—C6—C5	109.0 (2)	N1^iv^—Cd1—S2	90.73 (4)
O3—C6—H6A	109.9	N2^iii^—Cd1—S1^iii^	92.94 (4)
C5—C6—H6A	109.9	N1—Cd1—S1^iii^	172.93 (4)
O3—C6—H6B	109.9	N1^iv^—Cd1—S1^iii^	91.80 (4)
C5—C6—H6B	109.9	S2—Cd1—S1^iii^	83.363 (12)
H6A—C6—H6B	108.3	N2^iii^—Cd1—S1^v^	92.94 (4)
O3—C7—C8	109.5 (2)	N1—Cd1—S1^v^	91.80 (4)
O3—C7—H7A	109.8	N1^iv^—Cd1—S1^v^	172.93 (4)
C8—C7—H7A	109.8	S2—Cd1—S1^v^	83.363 (12)
O3—C7—H7B	109.8	S1^iii^—Cd1—S1^v^	91.373 (19)
C8—C7—H7B	109.8	C1—S1—Cd1^i^	98.27 (5)
H7A—C7—H7B	108.2	C2—S2—Cd1	93.24 (7)
			
O1—C3—C4—O2	63.7 (3)	C1—N1—Cd1—N2^iii^	107.3 (2)
O2—C5—C6—O3	−67.4 (2)	C1—N1—Cd1—N1^iv^	14.4 (2)
O3—C7—C8—O4	64.2 (3)	C1—N1—Cd1—S2	−76.3 (2)
C4—C3—O1—C3^ii^	177.23 (15)	C1—N1—Cd1—S1^v^	−159.7 (2)
C6—C5—O2—C4	178.6 (2)	N1—Cd1—S2—C2	42.19 (4)
C3—C4—O2—C5	−175.5 (2)	N1^iv^—Cd1—S2—C2	−42.19 (4)
C8—C7—O3—C6	176.27 (19)	S1^iii^—Cd1—S2—C2	−133.916 (9)
C5—C6—O3—C7	−178.75 (19)	S1^v^—Cd1—S2—C2	133.916 (9)
C7—C8—O4—C8^ii^	−179.93 (16)		

Symmetry codes: (i) −*x*+1, −*y*+2, *z*+1/2; (ii) −*x*+2, *y*, *z*; (iii) −*x*+1, −*y*+2, *z*−1/2; (iv) −*x*+1, *y*, *z*; (v) *x*, −*y*+2, *z*−1/2.

### Hydrogen-bond geometry (Å, º)

**Table:**

*D*—H···*A*	*D*—H	H···*A*	*D*···*A*	*D*—H···*A*
N3—H3*E*···O2	0.89 (1)	2.03 (1)	2.9130 (19)	174 (3)
N3—H3*D*···O4	0.90 (1)	2.05 (3)	2.892 (3)	155 (5)
